# Peanut sensitization pattern in Norwegian children and adults with specific IgE to peanut show age related differences

**DOI:** 10.1186/s13223-015-0095-8

**Published:** 2015-11-14

**Authors:** Ellen Namork, Berit A. Stensby

**Affiliations:** Division of Environmental Medicine, Department of Food, Water and Cosmetics, Norwegian Institute of Public Health, PO Box 4404, 0403 Oslo, Norway; Lovisenberggata 8, Oslo, Norway

**Keywords:** Peanut sensitization pattern, sIgE, Age related differences, Peanut allergens, Ara h 2, Ara h 8

## Abstract

**Background:**

Peanuts contain potent food allergens and the prevalence of allergy is reported to increase, especially in children. Since peanut sensitization may differ between different geographical regions, we wanted to investigate the sensitization pattern to the individual peanut allergens in a Norwegian population.

**Methods:**

Cases reported to the Norwegian Food Allergy Register with sera positive to peanut extract were analyzed for specific IgE (sIgE) to the recombinant peanut allergens Ara h 1, Ara h 2, Ara h 3, Ara h 8 and Ara h 9 and to birch pollen extract. Serum samples negative to the above allergens were analyzed for sIgE to Ara h 6, and sIgE to Pru p 3 in peach were analyzed in sera positive to the cross-reactive allergen Ara h 9.

**Results:**

Highest frequency of sIgE to Ara h 2, often co-sensitized to Ara h 1 and 3, were found in the small children up to 6 years of age. From the age of 6 years, sensitization to Ara h 8 was predominant. The sIgE levels to the storage proteins Ara h 1, 2 and 3 were strongly correlated, as was the sIgE levels to Ara h 8 and birch pollen extract. A low sensitization rate of sIgE to Ara h 9 in young adults was observed, which sIgE levels were very strongly correlated to Pru p 3.

**Conclusion:**

The sensitization to peanut allergens in a Norwegian population shows a clear age dependent pattern. The results add to the previously published research on the sensitization patterns of peanut sensitized patients in different geographical areas.

## Background

Peanut allergy represents a worldwide problem, it is often severe, potentially fatal and often persistent throughout life [1, 2]. The estimated prevalence of peanut allergy is between 0.5 and 2.0 % and appears to be increasing especially in children [3–5]. An accurate diagnosis of peanut allergy is essential since it may represent a significant burden on both quality of life and socio-economy [6]. Medically supervised oral food challenges (double-blind placebo-controlled food challenge, DBPCFC) are considered the gold standard for diagnosis but are resource-intensive and may be associated with risk of severe allergic reaction or anaphylaxis. In the last decades, however, several of the peanut allergens have been characterized, and analysis of specific IgE (sIgE) on a molecular basis has been evaluated as a diagnostic tool for peanut allergy [7–10].

The major peanut allergens Ara h 1, 2 and 3 belong to the seed storage proteins of the vicilin, conglutin and glycinin families, respectively, and are considered to be responsible for the original sensitization to peanut in susceptible individuals. The seed storage proteins are stable and associated with increased risk of severe reactions or anaphylaxis. The storage protein Ara h 6, a conglutin, has sequence identities to Ara h 2 and is also reported to be associated with clinical reactivity to peanut. [11]. The relationship between allergy to pollen and vegetables, nuts, peanuts and fruits is caused by cross-reacting epitopes due to homology between proteins and often give rise to milder symptoms such as the oral allergy syndrome. The peanut protein Ara h 8 is homologous to the birch pollen protein Bet v 1, and contributes to a substantial cross-reactivity between peanut and birch pollen [12]. Cross-reactivity between profilin in grass pollen and peanut may also occur [13]. The peanut allergen Ara h 9 is an enzyme-stable non-specific lipid transfer protein (LTP) with cross-reactive epitopes to other LTPs such as Pru p 3 in peach and Cor a 8 in hazelnut [14]. The protein Ara h 9 is reported to be an allergen of importance in the Mediterranean area that may cause systemic reactions in addition to oral allergy syndrome [15].

Recent studies have shown that peanut allergy in USA, Australia and different parts of Europe have different clinical and immunological patterns, due to differences in pollen exposures and differences in dietary traditions [13, 14, 16]. Since Norway is a birch endemic country and birch pollen gives rise to cross-reactions to peanut, we wanted to investigate the sensitization pattern to the individual peanut allergens in cases reported to the Norwegian National Reporting System and Register of Severe Allergic Reactions to Food (the Norwegian Food Allergy Register). The cases are submitted with serum samples routinely analyzed for a standard panel of allergen extracts [17]. All patients sensitized to peanut extract were analyzed for sIgE to the recombinant peanut allergens, in relation to age, gender, onset of reaction, symptoms and number of co-sensitizations to other foods and to birch pollen.

## Methods

### Patients

The Norwegian Food Allergy Register was established at the Norwegian Institute of Public Health in 2000 in collaboration with the Norwegian Food Safety Authority and the National Veterinary Institute [17]. Cases are reported on a voluntary basis by first-line doctors and submitted together with a serum sample. The reports contain patients’ information such as a short case history including gender, age, the suspected or incriminating food, and onset of reaction, known allergies, symptoms and the medication given. A written consent form is signed by all patients. A total of 1250 sera submitted to the Food Allery Register, routinely analyzed for sIgE to a panel of food allergens including peanut, birch- and timothy pollen were screened for sIgE to peanut extract. Two hundred and fourteen sera had sIgE antibodies to peanut extract above the cut off value 0.35 kU/l and were included in the study. The 214 patients were equally distributed between genders, 101 females and 113 males, and comprised ages from <1 to 80 years.

### Serological analysis

The patient sera were analyzed for sIgE using ImmunoCap^®^ (Phadia AB, Uppsala, Sweden). Due to limited volume of serum available for some of the patients, specific IgE antibodies to the three storage proteins Ara h 1, Ara h 2 and Ara h 3 were analyzed in 192 patient sera. IgE reactivity to Ara h 8, Ara h 9 and to birch pollen extract were analyzed in all 214 sera. Sera with sIgE to Ara h 9 were analyzed for sIgE to the peach allergen Pru p 3 known to result in cross-reactions to the lipid transfer protein. Sera with sIgE antibody levels >0.35 kU/l were considered positive. Since ImmunoCap with Ara h 6 is not commercially available, sera negative to all the above peanut allergens were analyzed for sIgE to Ara h 6 by ImmunoSorbent Allergen bioChip assay, ISAC (Thermo Fisher Scientific, Oslo, Norway), reported in standard units (ISU). ISU >0.3 were considered positive.

### Statistics

Pearson correlation was used to establish the strength of the relationship between sIgE antibody levels. Syntax of recoded combinations of sera with sIgE to the major allergens Ara 1, 2 and 3 made it possible to obtain the frequency of all combinations. Frequency analysis and plots of the collected data were made using the statistical programs IBM SPSS Statistics 22 and SigmaPlot 12.3.

## Results

### Gender, onset of reaction, symptoms and treatment in relation to age groups

**Fig. 1:**
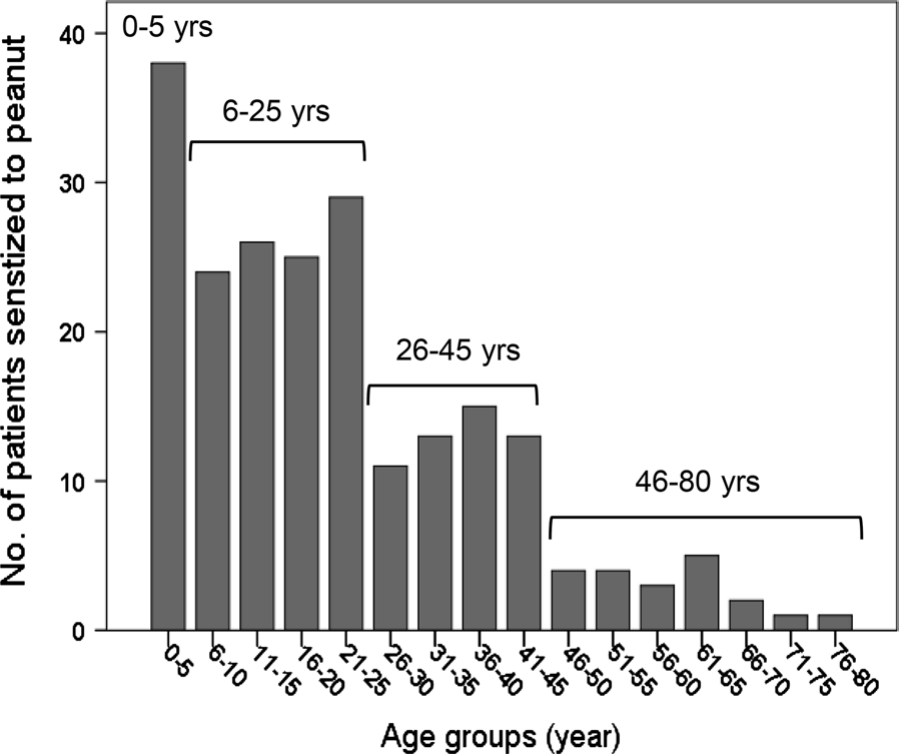
Age distribution of patients with peanut specific IgE show four age groups; 0–5 years, 6–25 years, 26–45 years and 46–80 years of age

Frequency analysis of the ages of the 214 patients sensitized to peanut showed four age groups (Fig. 1). As seen from the figure, the frequency of sensitization peaked at ages 0–5 years, the second group comprised the ages from 6 to 25 years, the third group from 26 to 45 years and the lowest frequency of sensitization was seen for the ages 46 to 80 years. The gender distribution shifted from 76:24 % males:females in the small children’s group to 23:77 % males:females in the oldest group (Table 1). The onset of reaction reported to occur within 1 h after intake of the suspected food was highest in the youngest group, 97 %, and decreased by age to 69 % in the oldest group (Table 1). The organ systems reported to be most often affected were symptoms in the skin (urticaria, and angioedema, sudden itching of eyes and nose), most often in combination with gastrointestinal tract (oral pruritus, lip swelling, abdominal pain, diarrhea, and vomiting) and/or respiratory symptoms (rhinorrhea, wheezing, chest tightness, cough, stridor, dyspnoe, and respiratory arrest). Patients reported to have symptoms affecting more than one organ system, which is related to high risk of severe reactions, were highest in the youngest age group, 76.7 %, declining with age to 54.5 % in the oldest age group (Table 1). The four age groups also differed in that severe skin symptoms were more often reported and loss of consciousness less reported in the youngest children. Cardiac arrest was not reported for the youngest and the oldest age groups. The therapeutic treatment employed was reported to be antihistamines alone (23.5 %), in combination with steroids (24.1 %) or most commonly in a combination of both epinephrine and steroids (32.1 %). Epinephrine alone and steroids alone was used in 14.2 and 6.2 % of the reported cases, respectively.

**Table 1:** Gender ratio, onset of reaction within 1 h and symptoms affected in more than one organ system reported in the 214 patients in relation to the four age groups

Age group	Reported date			ImmunoCap^®^ analysis		
Years	Gender ratio %	Onset of reaction %	Symptoms %	Ara h 2 %	Ara h 8 %	Birch pollen %
	Male:female	<1 h	>1 organ affected	sIgE >2.0 kU/l^b^	sIgE kU/l	sIgE kU/l
0–5 (N^a^ = 43)	76: 24	97	76.7	51.2	23.3	41.9
6–25 (N = 96)	49: 47	80	72.0	31.3	56.3	83.3
26–45 (N = 53)	26.5: 73.5	90	58.5	15.0	54.7	79.2
46–80 (N = 22)	23: 77	69	54.5	0.0	68.2	86.4

The patients sera analyzed for sIgE to Ara h 2 with levels >2.0 kU/l, sIgE to the birch pollen homologue Ara h 8 and birch pollen extract is also shown

^a^Number of patient sera

^b^Sera with sIgE to Ara h 2/6 >2.0 kU/l (marker for clinical allergy)

### Specific IgE levels to the peanut allergens

Seventy-four (38.5 %) of the 192 patient sera analyzed had sIgE to the three seed storage proteins Ara h 1, 2, and 3 in different combinations and sIgE co-sensitized to all three proteins was seen in 36 (48.7 %) patient sera. The proteins Ara h 1 and Ara h 2 were co-sensitized in 51 (68.9 %) of the patients, Ara h 2 and Ara h 3 in 39 (52.7 %) patients and Ara h 1 and Ara h 3 were co-sensitized in 38 (51.4 %) patients. Seventeen patients were mono-sensitized to Ara h2, Ara h1 and Ara h3 in frequencies of 9 (12.2 %), 6 (8.1 %) and 2 (2.7 %), respectively. The IgE levels to all three recombinant allergens were strongly correlated (r = 0.65–0.71, p < 0.01).

One hundred and eight (50.5 %) of the 214 patients sensitized to peanut extract had sIgE to the birch pollen homologue Ara h 8 and were co-sensitized to the birch pollen extract in all but two patients. Their sIgE levels were strongly correlated (r = 0.61, p < 0.01). Thirty-seven (34 %) of the patients with sIgE to Ara h 8 were co-sensitized to the three peanut storage proteins in different combinations and showed no correlation with respect to sIgE levels.

Twenty-four (11 %) of the 214 patients, showed to be sensitized to the lipid transfer protein Ara h 9 with co-sensitization to Pru p 3 with similar sIgE levels. The sIgE levels to the two allergens were very strongly correlated (r = 0.99, p < 0.01). Thirteen (54.2 %) of these patients showed co-sensitization in different combinations to Ara h 1, 2, 3 and 8.

Thirty-five (16.3 %) patient sera had no sIgE to any of the above peanut allergens and were analyzed for sIgE to the Ara h 2 homologue Ara h 6. Four (11.4 %) patients had sIgE to Ara h 6 with ISU values characterized as low (0.3 ISU), moderate to high (4.0 ISU and 7.9 ISU) and very high (20.0 ISU).

Thirty-one patient sera were negative to all six peanut allergens and had low levels of sIgE to peanut extract. These sera were all from patients between the ages 0–10 years and showed to have sIgE to birch- and/or timothy pollen and/or to seeds and nuts indicating cross-reaction to peanut due to primary sensitization to pollen or to seeds or nuts. Sensitization to peanut due to cross-reactivity between 2S albumins in nuts like walnut, and sesame seeds has been reported [18].

### Age related IgE profiles to peanut allergens

**Fig. 2:**
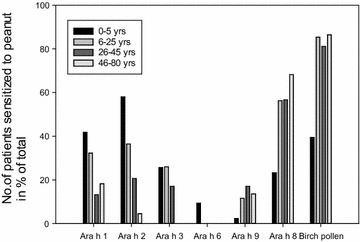
Sensitization pattern of specific IgE-antibodies to the peanut allergens and birch pollen extract in the four age groups showing highest frequency of sensitization to Ara h 2 in the youngest age groups, and highest frequency of sensitization to Ara h8 from the age of 6 years and in the older age groups

Specific IgE sensitization to the individual peanut allergens differed between the four age groups. Sensitization to the major storage proteins was highest in the youngest age groups and lowest in the oldest age group. The decrease according to age was especially marked with respect to Ara h 2 (Fig. 2). The youngest children (0–5 years) were most frequently sensitized to the seed storage protein Ara h 2 (58.0 %), but were frequently co-sensitized to Ara h 1 (44 %) and Ara h 3 (27 %) in different combinations. The four sera with sIgE to Ara h 6 were all from patients in the small children’s group. Sensitization to the lipid transfer protein Ara h 9 was seen in all age groups (only one positive in the children’s group) but most frequently among the young adults (26–45 years) (Fig. 2). The birch pollen homologue Ara h 8 increased markedly in frequency from 23.3 % in the youngest children to 56.3 % at the age of 6 years and was found to be 68.2 % in oldest age group. Similarly, sensitization to birch pollen showed a marked increase in frequency of sensitization from 41.9 % in the youngest children to 83.3 % at the age of 6 and showed similar high frequencies in the two older age groups (86.4 %) (Fig. 2). A level of sIgE to Ara h 2 > 2.0 kU/l, considered to be diagnostic for clinical peanut allergy [19], was found in 51.2 % of the sera from patients in the youngest age group. Sera with levels above this value decreased with age to 31.3 % in the second age group, 15 % in the third age group to none in the oldest age group (Table 1).

Co-sensitizations, to 1, 2 or 3 other food allergens or to more than 3 food allergens were equally common in all age groups. The most common sensitizations to allergens other than peanut were to other legumes, celery, wheat, seeds and tree nuts.

## Discussion

The pattern of sensitization to the six individual peanut allergens Ara h 1, 2, 3, 6, 8 and 9 was evaluated in patients reported to the Norwegian Food Allergy Register. Frequency analysis of the ages of the 214 patients sensitized to peanut showed four age groups; 0–5, 6–25, 26–45 and 46–80 years (Fig. 1). The sensitization pattern showed highest frequency of sIgE to the major peanut allergens Ara h 2/6, 1, and 3 in the youngest age group (58.5 %) and lowest frequency in the oldest age group (4.5 %), as opposed to the birch pollen homologue Ara h 8 which showed the highest frequency of sensitization in the oldest age group (68.2 %) and lowest frequency of sensitization in the youngest age group (23.3 %) (Fig. 2; Table 1).

Similar changes in gender distribution by age, as presently observed, from 76:24 % male:female in the youngest age group to the opposite ratio in the oldest group, has been reported for both asthma and allergy and is explained by hormonal changes, genetic susceptibility and differences in environmental exposure. The early onset of sensitization to the major peanut allergens and the early onset from intake of food to elicitation of symptoms in the children’s group, together with high incident of more than one organ system affected, indicate that the reactions were severe in these patients. Although the medical treatment of allergic reactions will vary between the individual doctors, all patients were treated with antihistamines, epinephrine and steroids or a combination of the three, indicating that the reactions were considered to be severe. Severity, however, based on the reports of symptoms, and the medication given is difficult to measure since it will depend on at which time course of reaction the patients were treated. An early onset of effective treatment will always be aimed at to avoid the most severe reactions which may explain why loss of consciousness and cardiac arrest was seldom reported.

The results showed the highest frequency of sensitization to Ara h 2, less to Ara h 1 and to a much less extent to Ara h 3 in the youngest children. Ara h 2 and Ara h 6 have been found to account for the majority of the effector activity in crude peanut extract [20], with equal diagnostic value [21] and hence, to be more potent than Ara h 1 and Ara h 3 [22]. However, co-sensitization to all 3 allergens has been shown to be correlated to severity of symptoms [23]. In the present study, sIgE to Ara h 6 was detected in four sera from the youngest children. Sensitization to Ara h 6 without concomitant sensitization to Ara h 2 was also reported in a Swedish study to be responsible for severe reactions [24]. In two of the present cases, sIgE levels to Ara h 6 were high (20.0 and 7.9 ISU) and the patients were reported to react with acute anaphylactic reaction after intake of one peanut. In the other two cases, however, with low sIgE levels to Ara h 6 (ISU 0.3 and 4.0), high levels of sIgE to cashew nut was detected. This may indicate primary sensitization to cashew nut with cross-reactivity to Ara h 6 due to sequence identity between storage proteins. The early onset of severe peanut allergy in children found in the present study is in line with findings in other population studies in children [7, 25, 26]. Further, in the studies comparing immunological differences among patients of different ages and in different geographical regions, early onset of sIgE to the three allergens Ara h 1, 2 and 3 were also reported, often presented with severe symptoms [13, 14, 16]. One may speculate if the high sensitization rate to the major peanut allergens in the youngest children is due to dietary changes with an increase in the overall use of peanuts in foods and as snacks over the last decades and/or as Ballmer-Weber et al. [16] speculates, an increase due to an intestinal permeability in genetically predisposed children. A recent study [27], however, showed that delayed oral exposure to peanut was associated with a greater frequency of clinical peanut allergy and hence may be responsible for the increased prevalence in this age group.

A study from Italy [28] reported no differences in sensitization among ages up to 16 years for the major peanut allergens, but reported increased levels of sIgE to Ara h 8 according to age, as found in the present study. The high correlation of sIgE levels to birch pollen extract and Ara h 8 may suggest primary pollen sensitization with following cross-reaction to Ara h 8, and possibly to other labile PR-10 proteins homologous to Bet v 1. The increase, however, in sensitization to birch pollen and Ara h 8 observed at the early age of 6 years, may, in part, be due to a milder climate and thereby longer pollen season [29]. Even if cross-reactions in general are considered to give milder reactions than sensitization to the major, stable allergens, the symptoms may have been experienced as severe and treated and reported as such. Thirty-seven (34 %) of the patients, however, with sIgE to Ara h 8 were co-sensitized to the three peanut storage proteins in different combinations, and may in these cases have been responsible for the severe reactions reported. Reactions caused by cross-sensitizations or co-sensitization to other food allergens than peanut cannot be ruled out.

All sera with sIgE to the lipid transfer protein Ara h 9, also had sIgE to Pru p 3 with similar sIgE levels and half of these patients were co-sensitized in different combinations to Ara h 1, 2, 3 and 8. All sera were in addition co-sensitized to other foods and often to hazelnut. The severe symptoms reported may, therefore, have been caused by sensitization to the major peanut allergens or by cross-reactions to LTP in hazelnut rather than to Ara h 9. The diagnostic value of Ara h 9 is said to be poor [19] and the clinical relevance of sensitization to Ara h 9 is difficult to interpret.

Various thresholds for sIgE to Ara h 2 have been suggested to predict clinically relevant peanut allergy but regional differences in addition to large individual variations make extrapolations between studies difficult [16]. The use of recombinant allergens, therefore, may be useful to distinguish patients with high risk of severe symptoms from those with less severe symptoms but cannot still replace oral challenges in determining thresholds and severity. Although the cases reported in the present study were submitted by first-line doctors who considered the reactions as being severe, the weaknesses of the study are that the results are based on cases with reported symptoms and serological analysis not verified by oral challenges. Hence, the overall information given including the severity of symptoms may have been biased by the reporting habits of the doctors. Further, the volume of the serum sample submitted, were in some cases small which limited the number of analysis. Still, the results from the submitted reports and the present analyses of peanut allergens in sensitized subjects, contribute to the information on peanut sensitization patterns in different populations.

## Conclusion

Component based analysis of peanut in patient sera from cases reported to the Norwegian Food Allergy Register sensitized to peanut, demonstrate a clear age dependent pattern. The early onset of sensitization to the main allergens Ara 1, 2 and 3 found in the children below the age of 6 years, showed highest frequency of sIgE to Ara h 2, indicating the importance of using Ara h 2 in diagnosing small children sensitized to peanut. The early debut of pollen sensitization, may be caused by warmer climate and longer pollen season and suggest a majority of primary sensitization to birch pollen from the age of 6 years, with following cross-sensitization to the birch pollen homologue Ara h 8 in peanut.
